# Supplementary material for: Comprehensive Analysis Identifies Ameloblastin-Related Competitive Endogenous RNA as a Prognostic Biomarker for Testicular Germ Cell Tumour
Source: Cancers (Basel). 2022 Apr 7;14(8):1870. doi: 10.3390/cancers14081870 (PMC9030878; doi:10.3390/cancers14081870)
Supplement: Supplementary file 1 [file cancers-14-01870-s001.zip › cancers-1639627-supplementary.pdf]

cancers

MDPI

Table S1. Baseline information sheet for AMBN

Tumour type	Number
Total	139
Embryonal carcinoma, NOS	26
Seminoma, NOS	68
Teratoma, benign	6
Teratoma, malignant, NOS	3
Mixed germ cell tumor	30
Yolk sac tumor	4
Teratocarcinoma	2

Table S2. Information of TGCT patients

Please find Table S2 in the separate .xlsx file.

Table S3. List of cancers analysed in the present study

ACC, Adrenocortical carcinoma

BLCA, Bladder Urothelial Carcinoma

BRCA, Breast invasive carcinoma

CESC, Cervical squamous cell carcinoma and endocervical adenocarcinoma

CHOL, Cholangiocarcinoma

COAD, Colon adenocarcinoma

DLBC, Lymphoid Neoplasm Diffuse Large B-cell Lymphoma

ESCA, Esophageal carcinoma

GBM, Glioblastoma multiforme

HNSC, Head and Neck squamous cell carcinoma

KICH, Kidney Chromophobe

KIRC, Kidney renal clear cell carcinoma

KIRP, Kidney renal papillary cell carcinoma

LAML, Acute Myeloid Leukemia

LGG, Lower Grade GLioma

LIHC, Liver hepatocellular carcinoma

LUAD, Lung adenocarcinoma

LUSC, Lung squamous cell carcinoma

MESO, Mesothelioma

OV, Ovarian serous cystadenocarcinoma

PAAD, Pancreatic adenocarcinoma

PCPG, Pheochromocytoma and Paraganglioma

PRAD, Prostate adenocarcinoma

READ, Rectum adenocarcinoma

SARC, Sarcoma

SKCM, Skin Cutaneous Melanoma

STAD, Stomach adenocarcinoma

TGCT, Testicular Germ Cell Tumor

THCA, Thyroid carcinoma

THYM, Thymoma

UCEC, Uterine Corpus Endometrial Carcinoma

UCS, Uterine Carcinosarcoma

UVM, Uveal Melanoma

Table S4. Single/multivariate Cox regression analysis report of GFAP

Characteristics	Total(N)	Univariate analysis		Multivariate analysis	
		Hazard ratio (95% CI)	P value	Hazard ratio (95% CI)	P value
GFAP	134				
Low	67	Reference			
High	67	1.261 (0.644-2.469)	0.500		
Pathologic stage	127				
Stage I	101	Reference			
Stage II&Stage III	26	0.244 (0.074-0.808)	0.021	41489858.955 (5529241.739-311328112.866)	<0.001

Characteristics	Total(N)	Univariate analysis		Multivariate analysis	
		Hazard ratio (95% CI)	P value	Hazard ratio (95% CI)	P value
Clinical stage	108				
Stage I	93	Reference			
Stage III	15	0.413 (0.125-1.365)	0.147		
Radiation therapy	132				
No	110	Reference			
Yes	22	0.738 (0.286-1.905)	0.530		
Primary therapy outcome	83				
PR&PD	14	Reference			
CR	69	7.730 (1.040-57.479)	0.046	91975579.183 (0.000-Inf)	0.998
Race	129				
Asian	4	Reference			
Black or African American	6	4.700 (0.485-45.582)	0.182	1328792526.067 (155125647.423-11382318827.779)	<0.001
White	119	1.043 (0.142-7.680)	0.967	179793514.854 (20989420.789-1540095284.601)	<0.001
Age	134				
<=30	64	Reference			
>30	70	0.713 (0.366-1.388)	0.320		
Serum tumor markers(S)	120				
S0	43	Reference			
S1	38	1.674 (0.646-4.337)	0.289		
S2	34	2.727 (1.094-6.794)	0.031		

Characteristics	Total(N)	Univariate analysis		Multivariate analysis	
		Hazard ratio (95% CI)	P value	Hazard ratio (95% CI)	P value
S3	5	3.980 (0.824-19.229)	0.086		
Lymphovascular invasion	130				
No	75	Reference			
Yes	55	1.553 (0.800-3.014)	0.194		
Testicular intratubular germ cell neoplasia	125				
Absent	68	Reference			
Present	57	0.742 (0.373-1.476)	0.395		
History of undescended testis	127				
No	104	Reference			
Yes	23	0.631 (0.244-1.630)	0.342		
Family history of testicular cancer	118				
No	104	Reference			
Yes	14	3.306 (1.474-7.416)	0.004	2.057 (0.758-5.585)	0.157
Laterality	129				
Left	72	Reference			
Right	57	1.236 (0.611-2.503)	0.556		
Pathologic T stage	133				
T1	76	Reference			
T2&T3	57	1.305 (0.672-2.535)	0.432		
Pathologic N stage	59				
N0	46	Reference			

Characteristics	Total(N)	Univariate analysis		Multivariate analysis	
		Hazard ratio (95% CI)	P value	Hazard ratio (95% CI)	P value
N1&N2	13	0.123 (0.017-0.916)	0.041	1.000 (0.133-7.504)	1.000
Pathologic M stage	119				
M0	115	Reference			
M1	4	0.000 (0.000-Inf)	0.996		
Clinical T stage	113				
T1	64	Reference			
T2&T3	49	0.918 (0.459-1.834)	0.808		
Clinical N stage	108				
N0	78	Reference			
N1&N2&N3	30	0.248 (0.086-0.718)	0.010	0.000 (0.000-0.000)	<0.001
Clinical M stage	128				
M0	120	Reference			
M1	8	0.723 (0.173-3.028)	0.657		

Table S5. Single/multivariate Cox regression analysis report of PELATON

Characteristics	Total(N)	Univariate analysis		Multivariate analysis	
		Hazard ratio (95% CI)	P value	Hazard ratio (95% CI)	P value
PELATON	134				
Low	67	Reference			
High	67	2.136 (1.073-4.250)	0.031	2.868 (1.102-7.462)	0.031
Pathologic stage	127				
Stage I	101	Reference			
Stage II&Stage III	26	0.244 (0.074-0.808)	0.021	3527424.573 (469578.360-26497652.318)	<0.001

Characteristics	Total(N)	Univariate analysis		Multivariate analysis	
		Hazard ratio (95% CI)	P value	Hazard ratio (95% CI)	P value
Clinical stage	108				
Stage I	93	Reference			
Stage III	15	0.413 (0.125-1.365)	0.147		
Radiation therapy	132				
No	110	Reference			
Yes	22	0.738 (0.286-1.905)	0.530		
Primary therapy outcome	83				
PR&PD	14	Reference			
CR	69	7.730 (1.040-57.479)	0.046	9667181.242 (0.000-Inf)	0.994
Race	129				
Asian	4	Reference			
Black or African American	6	4.700 (0.485-45.582)	0.182	100294585.597 (11692161.902-860320271.368)	<0.001
White	119	1.043 (0.142-7.680)	0.967	20643123.098 (2406538.096-177075331.538)	<0.001
Age	134				
<=30	64	Reference			
>30	70	0.713 (0.366-1.388)	0.320		
Serum tumor markers(S)	120				
S0	43	Reference			
S1	38	1.674 (0.646-4.337)	0.289		
S2	34	2.727 (1.094-6.794)	0.031		
S3	5	3.980 (0.824-19.229)	0.086		

Characteristics	Total(N)	Univariate analysis		Multivariate analysis	
		Hazard ratio (95% CI)	P value	Hazard ratio (95% CI)	P value
Lymphovascular invasion	130				
No	75	Reference			
Yes	55	1.553 (0.800-3.014)	0.194		
Testicular intratubular germ cell neoplasia	125				
Absent	68	Reference			
Present	57	0.742 (0.373-1.476)	0.395		
History of undescended testis	127				
No	104	Reference			
Yes	23	0.631 (0.244-1.630)	0.342		
Family history of testicular cancer	118				
No	104	Reference			
Yes	14	3.306 (1.474-7.416)	0.004	2.363 (0.864-6.461)	0.094
Laterality	129				
Left	72	Reference			
Right	57	1.236 (0.611-2.503)	0.556		
Pathologic T stage	133				
T1	76	Reference			
T2&T3	57	1.305 (0.672-2.535)	0.432		
Pathologic N stage	59				
N0	46	Reference			

**Characteristics**	**Total(N)**	**Univariate analysis**		**Multivariate analysis**	
		**Hazard ratio (95% CI)**	**P value**	**Hazard ratio (95% CI)**	**P value**
**N1&N2**	**13**	**0.123 (0.017-0.916)**	**0.041**	**1.000 (0.133-7.512)**	**1.000**
**Pathologic M stage**	**119**				
**M0**	**115**	**Reference**			
**M1**	**4**	**0.000 (0.000-Inf)**	**0.996**		
**Clinical T stage**	**113**				
**T1**	**64**	**Reference**			
**T2&T3**	**49**	**0.918 (0.459-1.834)**	**0.808**		
**Clinical N stage**	**108**				
**N0**	**78**	**Reference**			
**N1&N2&N3**	**30**	**0.248 (0.086-0.718)**	**0.010**	**0.000 (0.000-0.000)**	**<0.001**
**Clinical M stage**	**128**				
**M0**	**120**	**Reference**			
**M1**	**8**	**0.723 (0.173-3.028)**	**0.657**		

Table S6. Single gene logistics regression analysis report of PELATON

**Characteristics**	**Total(N)**	**Odds Ratio(OR)**	**P value**
**Pathologic stage (Stage II&Stage III vs. Stage I)**	**127**	**1.632 (0.684-4.050)**	**0.276**
**Clinical stage (Stage II&Stage III vs. Stage I)**	**125**	**1.938 (0.860-4.517)**	**0.115**
**Radiation therapy (Yes vs. No)**	**132**	**1.244 (0.496-3.178)**	**0.641**
**Primary therapy outcome (CR vs. PD&PR)**	**83**	**1.088 (0.342-3.619)**	**0.887**
**Age (>30 vs. <=30)**	**134**	**1.271 (0.645-2.516)**	**0.489**
**Lymphovascular invasion (Yes vs. No)**	**130**	**2.689 (1.320-5.611)**	**0.007**
**Testicular intratubular germ cell neoplasia (Present vs. Absent)**	**125**	**0.910 (0.449-1.843)**	**0.794**

Characteristics	Total(N)	Odds Ratio(OR)	P value
**History of undescended testis (Yes vs. No)**	**127**	**1.300 (0.525-3.299)**	**0.572**
**Family history of testicular cancer (Yes vs. No)**	**118**	**0.495 (0.144-1.533)**	**0.234**
**Laterality (Right vs. Left)**	**129**	**0.417 (0.202-0.844)**	**0.016**
