# Supplementary figures and images for: Comprehensive Analysis Identifies Ameloblastin-Related Competitive Endogenous RNA as a Prognostic Biomarker for Testicular Germ Cell Tumour
Source: Cancers (Basel). 2022 Apr 7;14(8):1870. doi: 10.3390/cancers14081870 (PMC9030878; doi:10.3390/cancers14081870)

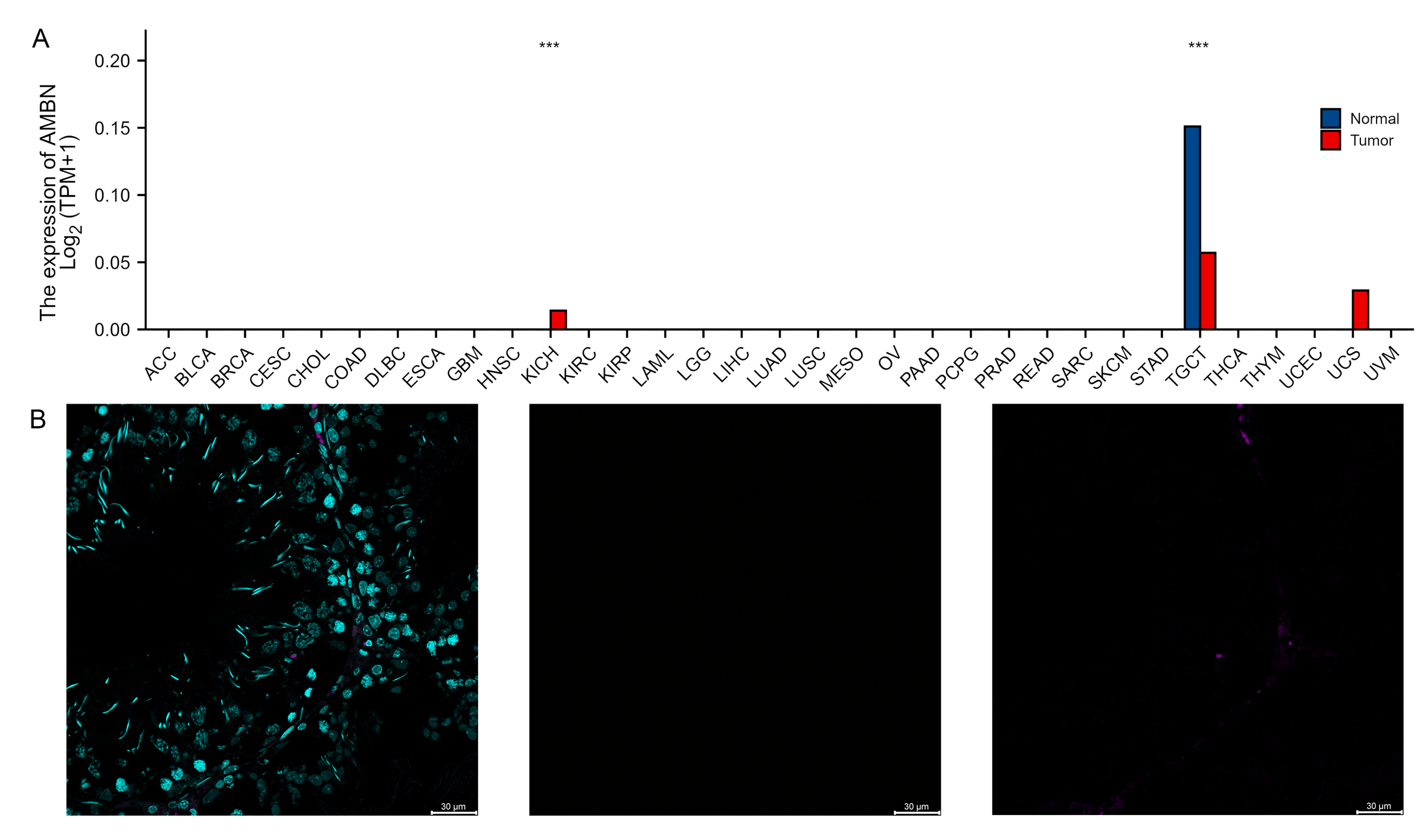

Supplement: Supplementary file 1 [file cancers-14-01870-s001.zip › supplement Figure S1.tif]

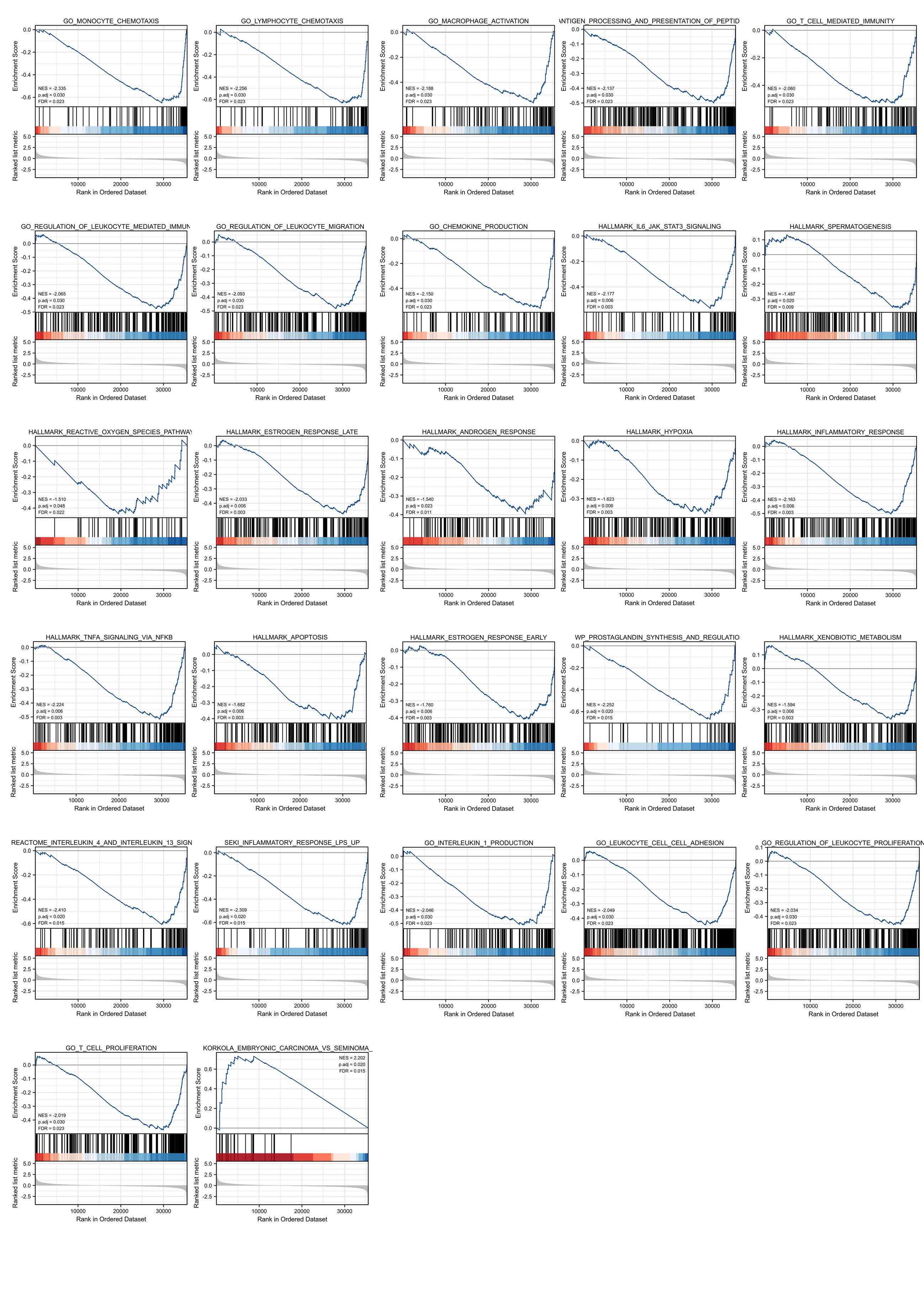

Supplement: Supplementary file 1 [file cancers-14-01870-s001.zip › supplement Figure S2.tif]
